# Ischémie aiguë du membre supérieur: complication d’un syndrome coronarien aigu (à propos d’un cas)

**DOI:** 10.11604/pamj.2026.54.7.50791

**Published:** 2026-05-11

**Authors:** Dialtabé Ibrahima Guessé Ba, Abdou Lahat Khouma, Mohamadou Al Khalifa Ba, Eliane Somyarian Sawadogo, Ousmane Ndiaye, Papa Arona Sene

**Affiliations:** 1 Service de Chirurgie Thoracique et Cardiovasculaire, Centre Hospitalier Régional Heinrich Lubke, Diourbel, Sénégal,; 2 Service de Cardiologie, Centre Hospitalier Régional Heinrich Lubke, Diourbel, Sénégal,; 3 Service d'Anesthésie-Réanimation, Centre Hospitalier Régional Heinrich Lubke, Diourbel, Sénégal

**Keywords:** Ischémie aiguë du membre, syndrome coronarien aigu, thrombectomie, cas clinique, Acute limb ischemia, acute coronary syndrome, thrombectomy, case report

## Abstract

Nous rapportons ici le cas d'une femme de 30 ans souffrant d'un syndrome coronarien aigu compliqué d'une ischémie aiguë du membre supérieur gauche. Elle a bénéficié d'une thrombectomie à la sonde de Fogarty par abord huméral avec des suites opératoires simples. Étant donné la rareté de l'ischémie aiguë non traumatique du membre supérieur, ce cas souligne l'importance du diagnostic et de la prise en charge des syndromes coronariens aigus pour éviter d'éventuelles complications qui menacent le pronostic fonctionnel et vital.

## Abstract (en)

We here report the case of a 30-year-old woman presenting with acute coronary syndrome complicated by acute ischemia of the left upper limb. She underwent Fogarty thrombectomy via humeral approach, with uncomplicated postoperative course. Given the rarity of non-traumatic acute upper limb ischemia, this case highlights the importance of diagnosis and management of acute coronary syndromes to prevent potential complications that may threaten both functional and vital prognosis.

## Introduction

L'ischémie aiguë du membre est une urgence médico-chirurgicale. Sa localisation au membre supérieur est rare. Le syndrome coronarien aigu en est une étiologie avec un mécanisme physiopathologique très bien élucidé. L'évolution du syndrome coronarien aigu est imprévisible avec un risque d'ischémie du membre, d'accident vasculaire cérébral engageant le pronostic fonctionnel et vital. La rareté de l'ischémie aiguë du membre supérieur mais aussi son étiologie (syndrome coronarien aigu) font la particularité de ce cas.

## Patient et observation

**Information sur la patiente:** la patiente YF, âgée de 32 ans, a été reçue aux urgences du Centre Hospitalier Régional Heinrich Lübke de Diourbel (CHRHLD) pour une ischémie aiguë du membre supérieur gauche compliquant un syndrome coronarien aigu. La patiente aurait présenté 3 jours auparavant une douleur angineuse, rétrosternale, intense avant l'apparition de la douleur vive au membre supérieur gauche s'accompagnant rapidement d'une impotence fonctionnelle absolue surtout des deux premiers doigts et de froideur du membre. Il n'y a pas d'antécédents personnels ni familiaux rapportés ni de traitement antérieur.

**Figure 1:**
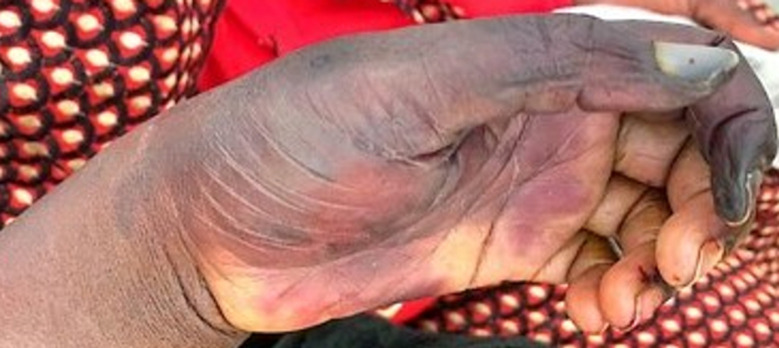
trouble trophique de la main gauche

**Résultats cliniques:** l'examen clinique notait un état général passable; les constantes hémodynamiques étaient normales; il y avait un syndrome d'ischémie aiguë du membre supérieur gauche (Figure 1): momification et cyanose des deux premiers doigts remontant à l'éminence thénarienne, sécheresse cutanée, refroidissement cutané, diminution de la sensibilité des premiers doigts, une abolition des pouls radial et ulnaire.

**Figure 2:**
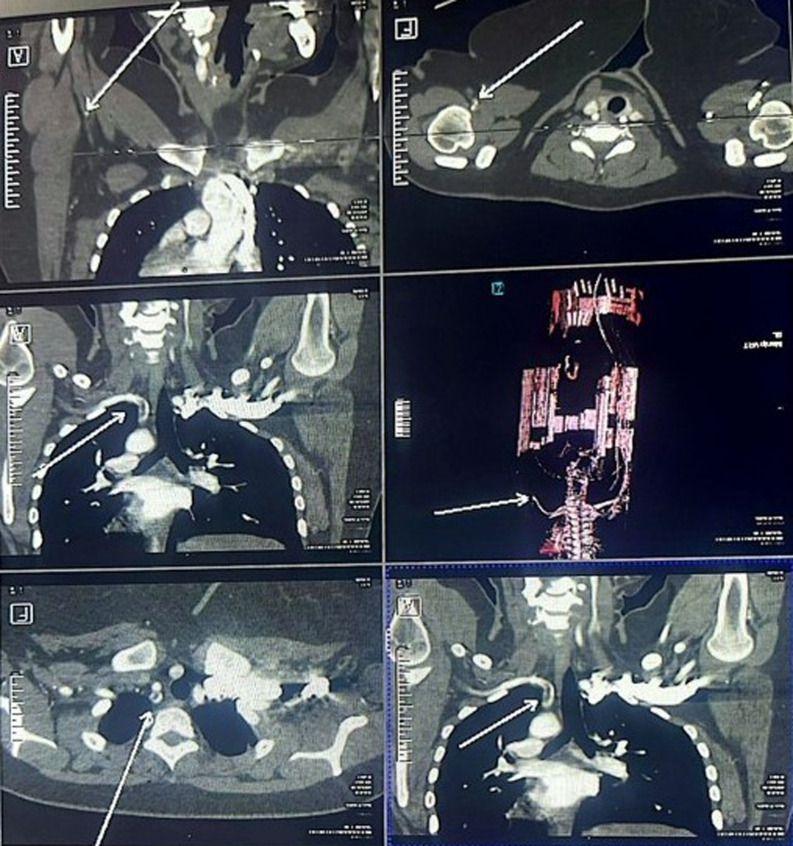
angio-TDM montrant le thrombus depuis l'artère subclavière gauche

**Figure 3:**
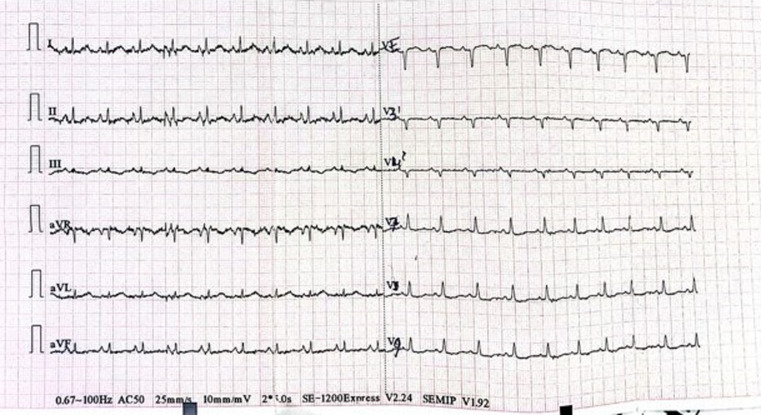
électrocardiogramme de la patiente

**Démarche diagnostique:** l'angio-TDM du membre supérieur (Figure 2) montre la présence d'un thrombus partiellement flottant à l'origine de la crosse de l'artère sous-clavière gauche avec détachement distal obstruant les artères axillaire et humérale proximale et une faible reprise en distalité avec revascularisation médiocre de l'arcade artérielle palmaire superficielle et profonde. A la recherche d'étiologie, on retrouve en regard de l'apex cardiaque, une formation hypodense de 18,63×16mm évoquant un thrombus pouvant en être à l'origine. Le bilan cardiaque réalisé montre à l'ECG (Figure 3) une tachycardie sinusale régulière avec fréquence cardiaque à 114cpm, axe QRS à +50, PR fixe à 16/100ms, QRS fins, lésion-nécrose en antéro-septal, extrême sans particularité. L'écho-Doppler cardiaque montre des cavités cardiaques de taille normale avec présence d'un thrombus apical de 19x14mm. Akinesie de la couronne apicale, hypocinésie des parois antéro-septales et inféro-septales. Hypercinésie compensatrice des autres parois. Fonction systolique du VG à 50% au Simpson biplan. Les résultats des explorations biologiques notaient: une hyperleucocytose à (25500/µL); créatininémie (11,7 umol/l); plaquettes (333000/µL) ; troponémie (58ng/L). Le diagnostic d'un syndrome coronarien aigu compliquant une ischémie aiguë du membre supérieur gauche a été retenu.

**Figure 4:**
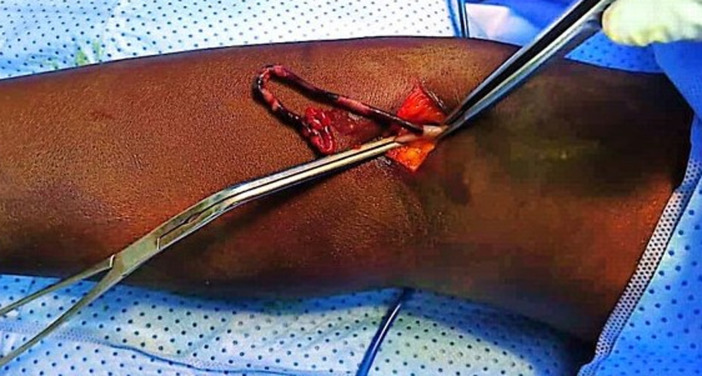
abord huméral montrant le thrombus

**Intervention thérapeutique et suivi:** il a été indiqué et réalisé une thrombectomie à la sonde de Fogarty par abord huméral (Figure 4). Les suites opératoires ont été simples (régression puis disparition de la douleur, réapparition de la chaleur locale, conservation de la motricité et de la sensibilité des trois derniers doigts, mais persistance de la momification des deux premiers doigts et de l'éminence thénar). Les examens biologiques se sont normalisés. La patiente est sortie à J2 post-opératoire sous triple anticoagulation (clopidogrel, rivaroxaban, aspirine) et bisoprolol et un suivi cardiologique. La cicatrisation de l'abord a été obtenue au 14^e^ jour post-opératoire.

**Perspective de la patiente:** la patiente était satisfaite des traitements reçus à l'hôpital surtout après la chirurgie qui a fait disparaître la douleur ischémique.

**Consentement de la patiente:** le consentement éclairé de la patiente a été obtenu.

## Discussion

L'ischémie aiguë du membre supérieur est une entité rare. L'originalité de notre observation réside dans l'étiologie de cette ischémie, le syndrome coronarien aigu. La proportion d'ischémie du membre supérieur était de 17,7%. L'incidence annuelle de l'ischémie aiguë du membre supérieur traitée chirurgicalement était de 1,3 cas pour 100 000 par an au cours des 15 dernières années d'étude [1].

Le syndrome coronarien aigu est une cause importante de morbidité et de mortalité au Sénégal. Les tendances démographiques nationales des hospitalisations pour infarctus du myocarde montrent une prévalence globale de 9% au Sénégal. À Dakar, la prévalence a augmenté avec une différence significative entre les périodes (1998 et 2010) et (2013 et 2020) passant de 6% à 13%. La proportion de patients présentant un SCA ST+ variait entre 44 et 94%. L'âge moyen des patients variait entre 57,1 et 64 ans [2]. Sur le plan physiopathologique, l'hémodynamique du ventricule gauche est significativement affectée pendant et après un infarctus aigu du myocarde. Après une lésion du myocarde, la contractilité cardiaque et la compliance ventriculaire sont significativement impactées. La relation pression-volume en fin de systolique, qui est corrélée à la contractilité cardiaque, est fortement réduite. En conséquence, la génération de pression diminue et le volume de la course aussi. La relation pression-volume en fin de diastolie, qui est corrélée à la compliance ventriculaire, connaît également des changements. La courbe de compliance se déplace, le ventricule gauche devenant plus rigide et moins docile. En conséquence, on observe une réduction du travail des AVC cardiaques, et donc du travail mécanique cardiaque global, ce qui est préjudiciable à la perfusion des organes s'il persiste [3].

Le déchargement du ventricule gauche peut aider à résoudre ces défis hémodynamiques qui, dans l'ensemble, réduisent le remodelage cardiaque et la taille de l'infarctus [3]. Ce qui peut contribuer à éviter le bas débit cardiaque et la formation de thrombus à l'origine de l'ischémie aiguë du membre. En effet, la patiente nous a été adressée trois jours après le début des symptômes. Un diagnostic précoce aurait certainement permis d'améliorer davantage le pronostic par un geste de revascularisation du membre (thrombectomie) et l'administration en urgence d'anticoagulants et/ou de thrombolytiques. Les résultats cliniques dans l'ischémie causée par une embolie sont nettement meilleurs que ceux de la thrombose locale [1]. Du point de vue pronostique, la fréquence de l'insuffisance cardiaque du syndrome coronarien aigu au Sénégal varie entre 6,7 et 52,9% et celle du choc cardiogénique entre 1 et 18,5%. La létalité globale était de 10% [2].

## Conclusion

Le cas rapporté ici pose le problème de la prise en charge des syndromes coronariens aigus (SCA) qui demeure problématique au Sénégal, malgré des avancées notables. Toutefois, le diagnostic précoce et l'amélioration de la prise en charge des syndromes coronariens aigus sont plus que jamais nécessaires pour améliorer davantage la morbi-mortalité et éviter d'éventuelles complications emboliques qui engagent le pronostic fonctionnel et vital.

## References

[1] Dubouis A, Vernier-Mosca M, Rinckenbach S, Salomon Du Mont L (2021). Results of the Surgical Management of Acute Limb Ischemia in the Nonagenarians. Ann Vasc Surg.

[2] Papa MG, Dioum M, Cherif MM, Diack B, Kane A (2024). Épidémiologie, prise en charge et pronostic des syndromes coronariens aigus au Sénégal: revue systématique de la littérature grise avec méta-analyse de 1998 à 2020. Annales de Cardiologie et d'Angéiologie.

[3] Upadhyaya VD, Wong C, Zakir RM, Aghili N, Faraz H, Kapur NK (2024). Management of Myocardial Infarction: Emerging Paradigms for the Future. Methodist Debakey Cardiovasc J.

